# Advancing Sustainable HIV Services Through Integration in Primary Healthcare in Sub-Saharan Africa: A Perspective on Practical Recommendations

**DOI:** 10.3390/healthcare13020192

**Published:** 2025-01-19

**Authors:** Tafadzwa Dzinamarira, Gallican Rwibasira, Loveday Mwila, Enos Moyo, Derek Mangoya, Perseverance Moyo, Edward Oladele, Adewale Akinjeji, Munashe Chimene, Claude Mambo Muvunyi

**Affiliations:** 1 School of Health Systems and Public Health, Faculty of Health Sciences, University of Pretoria, Pretoria 0002, South Africa; 2 ICAP in Zambia, Lusaka, Zambia; 3 Rwanda Biomedical Center, Kigali P.O. Box 7162, Rwanda; 4 Ministry of Health, Lusaka 10101, Zambia; 5 Department of Public Health Medicine, University of KwaZulu Natal, Durban 4041, South Africa; 6 The Centre for HIV and AIDS Prevention Studies, Windhoek 9000, Namibia; 7 Medical Center Oshakati, Oshakati, Namibia; 8 FHI 360, Lusaka, Zambia; edward.a.oladele@gmail.com; 9 Faculty of Public Health, College of Medicine, University of Ibadan, Ibadan, Nigeria; 10 Health Systems and Policy Research Group, Department of Global Public Health, Karolinska Institutet, 171 77 Solna, Sweden; 11 Ministry of Health and Child Care, Harare P.O. Box CY 573, Zimbabwe

**Keywords:** HIV, primary healthcare, health service integration, sustainability

## Abstract

Sub-Saharan Africa continues to bear a disproportionate burden of the global HIV epidemic. Integrating HIV services into primary healthcare is a crucial strategy to accelerate progress towards ending the epidemic. However, several challenges hinder effective integration, including underfunding, human resource shortages, infrastructure limitations, weak health systems, and sociocultural factors. With this perspective, we discuss strategies to address these challenges. A comprehensive, multi-faceted approach is necessary to overcome these barriers. Investing in human resources, improving infrastructure, and strengthening health information systems are essential for strengthening the health system. Implementing patient-centered care, integrated service delivery models, and community engagement can optimize service delivery. Utilizing digital health tools, such as mobile health applications and electronic health records, can enhance service delivery and data management. Mobilizing for an increase in domestic resources, aligning donor funding, and using cost-effective approaches are crucial for effective financing. Finally, robust monitoring and evaluation systems are necessary to track progress, identify challenges, and inform decision-making. With these strategies, among many others, sub-Saharan African countries can significantly improve the integration of HIV services into primary healthcare, leading to better health outcomes for people living with HIV and more sustainable HIV programs.

## 1. Introduction

The human immunodeficiency virus (HIV), responsible for acquired immune deficiency syndrome (AIDS), is thought to have been transmitted to humans from apes via exposure to infected blood and/or body fluids [1]. The deterioration of the cellular immune system characterizes HIV infection. CD4+ T cell counts decline progressively over time, and this reduction is linked to the onset of AIDS in individuals with HIV who do not receive antiretroviral therapy (ART) [2]. The World Health Organization classifies HIV into four clinical stages. During stage 1, people living with HIV (PLHIV) exhibit no symptoms. In stage 2, PLHIV exhibit mild symptoms, including unexplained weight loss. In stage 3, they display more severe symptoms, such as pulmonary tuberculosis (PTB). In the final stage, individuals exhibit AIDS-defining symptoms such as HIV encephalopathy, extrapulmonary tuberculosis, or Kaposi’s sarcoma [3]. HIV diagnosis typically involves the use of both antibody and antigen tests. The standard initial HIV treatment regimen comprises three antiretroviral medications from at least two distinct drug classes [4].

Sub-Saharan Africa (SSA) continues to disproportionately bear the burden of the global HIV epidemic, with high prevalence rates and substantial mortality, despite treatment progress. In 2023, approximately 40 million individuals were living with HIV, with around 65% residing in SSA. In 2023, there were 1.3 million new infections globally, with 640,000 occurring in SSA. In 2023, women and girls represented 44% of all new infections globally, whereas in SSA, this figure rose to 62%. This differs from other geographical regions, where more than 73% of new infections occurred among boys and men [5]. Over the past few decades, SSA has seen substantial improvements in the HIV response, such as increased access to antiretroviral therapy (ART), improved viral suppression, and a reduction in AIDS-related deaths. These achievements are the result of concerted efforts, including expanded HIV testing, the scaling up of ART programs, and the integration of HIV care into broader healthcare services [6]. Integrated HIV services in primary healthcare settings have shown notable successes, including increased HIV testing uptake, higher initiation rates of ART, improved retention in HIV care, and better outcomes in viral suppression in integrated service settings [7]. Furthermore, the treatment success of non-HIV-related conditions has also been enhanced through the integration of services [8].

Despite these gains, significant challenges remain in achieving the ambitious goal of ending the HIV epidemic by 2030. The lack of a vaccine presents a significant challenge in the effort to end the HIV epidemic. The challenges in developing an HIV vaccine stem from several factors, including the virus’s high mutation rates during replication, its significant conformational adaptability, and the extensive glycan shielding of the viral envelope, which allows it to evade neutralizing antibodies and other immune responses [9]. The initial trial of an HIV-1 vaccine in sub-Saharan Africa was carried out in Uganda utilizing a live recombinant canarypox vector [10]. Another trial was conducted in Southern Africa using a heterologous HIV-1 vaccine regimen consisting of a tetravalent mosaic adenovirus 26-based vaccine (Ad26.Mos4.HIV) and aluminum phosphate-adjuvanted clade C glycoprotein (GP) 140 in young women at risk of acquiring HIV-1 [11]. None of the potential vaccines have shown efficacy in preventing HIV acquisition. Additionally, in the face of dwindling global health funding, complex political dynamics in the Global North, rising inflation, and reduced national government health budgets, there is a pressing need to explore more sustainable models for implementing HIV programs across the region. Achieving the goal of ending the epidemic will require continued innovation, strategic investments, and the scaling of successful integrated service models. While there is a growing consensus on the importance of integrating HIV services into primary healthcare, there is a critical need for more research on effective implementation strategies and practical guidance to support their adoption in SSA.

Primary healthcare serves as the foundation of a robust health system. Its proximity to communities and its capacity to provide essential health services make it an ideal platform for delivering HIV prevention, testing, treatment, care, and support. It has been well established that countries can enhance access, improve efficiency, and optimize resource utilization by integrating HIV services into primary care [7]. However, translating the concept of integration into actionable steps has proven challenging.

A complex interplay of domestic and external resources characterizes the financial landscape for HIV and primary healthcare in SSA. Government funding, while often insufficient, forms the backbone of healthcare financing. While funding often skews towards specific disease programs, such as HIV, at the expense of primary healthcare, this imbalance can hinder the integration of HIV services and the provision of comprehensive care [12]. Donor funding is crucial in supporting HIV and health programs in the region. While this support has been instrumental in scaling up HIV treatment and prevention services, it often comes with specific conditions and time-bound commitments. This can create challenges regarding sustainability and integration with domestic financing mechanisms. Additionally, the fragmentation of donor funding across various health programs can lead to inefficiencies and the duplication of efforts [13].

Out-of-pocket payments remain a significant source of healthcare financing for many countries in SSA. However, these payments can pose a severe financial burden on households, particularly those living in poverty. Furthermore, out-of-pocket payments can deter people from seeking care, including HIV services, due to financial constraints [12]. While this paper does not delve into specific health financing data, understanding these financial constraints is crucial for developing practical recommendations in the context of integration.

Although regional and international bodies have issued guidance on integrating HIV services into primary healthcare, there is a dearth of concrete recommendations for implementation in SSA. In this perspective, we draw on existing research, case studies, and expert opinions to examine the challenges faced in primary healthcare settings within the region. We will discuss evidence-based approaches for overcoming these challenges and offer practical strategies informed by empirical data and real-world experiences. We aim to provide actionable recommendations for strengthening the integration of HIV services into primary healthcare in SSA based on a review of the current literature and successful implementation models.

**Figure 1:**
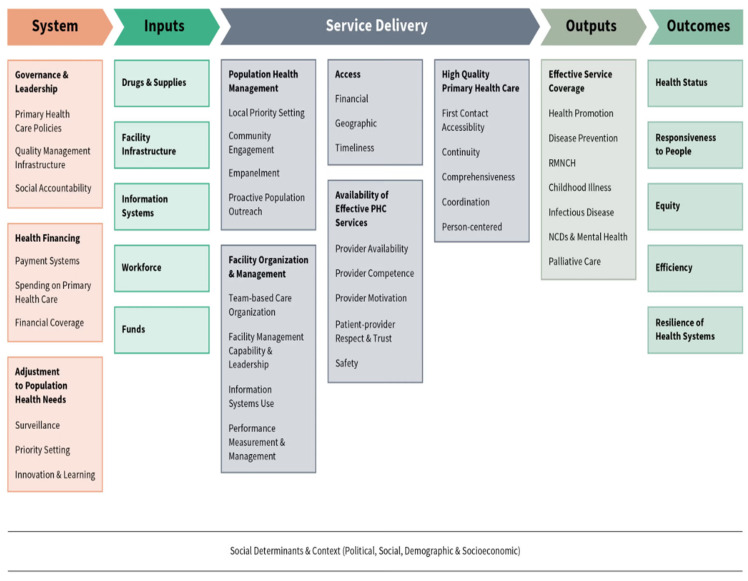
The Primary Healthcare Performance Initiative (PHCPI) Conceptual Framework (available at https://www.improvingphc.org/phcpi-conceptual-framework, accessed on 1 December 2024).

To guide our perspective, we draw on two key frameworks that address this complex issue’s conceptual underpinnings and implementation strategies. The Primary Healthcare Performance Initiative (PHCPI) Conceptual Framework (Figure 1) provides an analytic lens through which we understand the broader context of primary healthcare and how its strengthening can facilitate the integration of HIV services [14]. This framework emphasizes key domains such as governance, financing, health systems, and adjustments to population needs, which are critical for ensuring that HIV services are effectively integrated into primary healthcare. Additionally, the UNAIDS HIV Response Sustainability Primer offers essential guidance on creating sustainable HIV programs, focusing on elements such as financing models, governance structures, and the long-term viability of HIV interventions [15]. We use these two frameworks to inform the strategic approaches to strengthening primary healthcare in the region and the practical implementation steps necessary for integrating HIV services. The PHCPI framework guides our discussion on systemic improvements, while the UNAIDS primer focuses more specifically on the sustainability of HIV programs and the long-term effectiveness of integration efforts.

## 2. Understanding the Context: Challenges in Primary Healthcare That Limit HIV Integration

Significant disparities in access, quality, and coverage characterize primary healthcare in SSA. Remote and marginalized populations often bear the brunt of these inequities, with the limited availability of essential health services. A wide range of difficulties that jeopardize the effectiveness of primary healthcare systems further exacerbate this situation [16].

### 2.1. Underfunding

The chronic underfunding of healthcare, especially primary healthcare, remains pervasive across the region [13]. Government allocations for this critical level of care often fall short of recommended levels [17,18], leading to inadequate infrastructure, equipment, and human resources. This financial constraint severely limits the capacity of primary healthcare facilities to provide comprehensive services, including essential HIV prevention, testing, and treatment. Several studies, including one conducted in Kenya [19], revealed that more funding was required to ensure that primary healthcare services function optimally. Of concern, more than one in eight countries in the region spend more on debt servicing than they do on their own health budgets, highlighting the stark imbalance in resource allocation and the dire need for increased domestic and international investment in health systems. According to the Institute for Health Metrics and Evaluation [20], this trend underscores the urgent need for sustained financial commitments to health systems, particularly in areas like HIV care, where the need for resources is critical. Moreover, the reliance on donor funding for a substantial portion of health budgets can lead to unpredictable resource flows, hindering long-term planning and sustainability [12]. The instability and limitations of donor-driven funding further underscore the need for a more robust and diversified financing strategy to ensure the continued success and sustainability of HIV programs.

### 2.2. Human Resources for Health

The acute shortage of healthcare workers, particularly in remote areas, significantly hinders the delivery of quality primary healthcare. A study conducted in five selected countries to represent all the regions in SSA revealed that few doctors, nurses, and midwives are working in primary healthcare, and shortages of qualified staff are greatest in rural areas [21]. Poor working conditions, low pay, challenging living conditions, and few opportunities for professional development are just a few factors that make this deficit worse. Consequently, healthcare facilities are often understaffed, leading to overburdened healthcare providers and compromised patient care [22]. This lack of skilled personnel, including doctors, nurses, and community health workers, coupled with the growing population’s needs, hamper the effective integration of HIV services into primary care.

### 2.3. Infrastructure Limitations

Inadequate infrastructure is another critical challenge facing primary healthcare in SSA. Many facilities lack essential amenities such as electricity, clean water, and proper sanitation, creating an environment conducive to the spread of infections. The absence of adequate storage facilities for medications, including antiretroviral drugs, can compromise the quality of care and patient outcomes. A study conducted in Tanzania revealed serious infrastructural deficiencies in primary healthcare facilities [23]. Furthermore, poor transportation networks hinder access to healthcare services, particularly in remote areas, limiting the reach of HIV prevention and treatment programs.

### 2.4. Weak Health Systems

Fragile health systems characterized by poor governance, corruption, and weak accountability further impede the delivery of primary healthcare services. Inefficient management, lack of coordination among different levels of care, and limited data systems hinder the effective allocation of resources and the monitoring of health outcomes [24]. These systemic weaknesses create an environment where HIV service integration is difficult to achieve and sustain.

### 2.5. Sociocultural Factors

Sociocultural norms, beliefs, and practices significantly influence care-seeking behaviors and the uptake of services in primary healthcare, including HIV services. In many communities, traditional and religious beliefs intersect with perceptions of health and illness, affecting individuals’ willingness to seek care or adhere to treatment regimens. In particular, sociocultural factors such as stigma, gender roles, and taboos surrounding sexual health can create significant barriers to the integration of HIV services into primary healthcare. For example, stigma associated with HIV and other sexually transmitted infections (STIs) can discourage individuals from accessing integrated services, as they may fear judgment or discrimination from healthcare providers or their communities. This is especially true for populations at higher risk, such as women, adolescents, and men who have sex with men, who may already face societal marginalization. In some regions, cultural norms, such as those related to gender roles and sexual behavior, can hinder the adoption of preventive measures and the uptake of HIV services. In Kenya, cultural beliefs and practices, such as early marriage and polygamy, have been linked to the high prevalence of HIV among young women [25], creating challenges in both prevention and care.

## 3. Approaches to Addressing Challenges in Primary Healthcare for Successful HIV Integration

The interconnected challenges in primary healthcare provision underscore the complexities involved in strengthening it in sub-Saharan Africa. Overcoming these obstacles requires a multi-faceted approach that addresses the root causes of the problem and invests in building resilient health systems. The Primary Healthcare Performance Initiative (PHCPI) Conceptual Framework provides an essential lens for understanding how strengthening primary healthcare can facilitate the integration of HIV services. The PHCPI framework emphasizes the importance of key health system functions, including financing, governance, service delivery, and workforce development. In this section, using some framework concepts, we discuss practical solutions to the challenges faced by health systems in SSA and provide actionable recommendations that directly address the integration of HIV services into primary healthcare (PHC). Strengthening PHC through evidence-based policy and financing reforms, human resource capacity building, and adopting innovative service delivery models can help ensure that HIV services are better integrated, accessible, and sustainable across the region. Table 1 presents general recommendations for integrating HIV services into PHC, addressing policy, governance, and overall strategy at the national level.

### 3.1. Financing Integration and Allocating Resources Effectively

The effective integration of HIV services into primary healthcare in SSA necessitates a robust financial foundation. While significant progress has been made in the fight against HIV, persistent funding gaps and inefficient resource allocation continue to hamper progress. To ensure adequate and sustainable financing for HIV service integration, a diversified approach is required. Innovative financing mechanisms can complement traditional funding sources and optimize resource utilization.

#### 3.1.1. Domestic Resource Mobilization

Despite the persistent challenges, several countries in SSA have demonstrated innovative approaches to health financing that can guide future efforts. For instance, for the past two years, Botswana has allocated 17% of the government budget to the Ministry of Health and Wellness, the country’s second-highest budget allocation. The country’s public care is characterized by a primary healthcare model, with health posts and clinics comprising 95% of government health facilities. This budget allocation supports results in Botswana funding more than 60% of its health needs [26]. Similarly, Rwanda has implemented community-based health insurance schemes that have expanded coverage for underserved populations, including people living with HIV. These models underscore the importance of political commitment and community involvement in creating sustainable financing mechanisms.

While some countries have demonstrated innovative health financing approaches, much of the funding in SSA still remains centralized or fragmented, often not reaching the specific needs of PHC integration. To drive meaningful change, subnational governments (local, provincial, or state authorities) must allocate specific funding for HIV services within the PHC budget. Allocating dedicated HIV funding within the PHC framework can facilitate targeted resource allocation, improve service delivery, and ensure that PHC facilities are adequately supported to provide comprehensive HIV prevention, testing, and treatment services. This includes leveraging public–private partnerships and aligning donor funding with PHC-specific needs, ensuring that HIV service integration is sustainable and locally responsive.

Progressive taxation, improved revenue collection, and efficient expenditure management have also increased domestic financial resources for health. Taxes on different items such as alcohol, tobacco products, money transfers, mobile phone usage or mobile operators, and sugar-sweetened beverages have been used in different countries in SSA. Although the revenue raised for single items was low, it was observed that combining different items or services resulted in an increase in revenue raised for healthcare [27]. In Zimbabwe, a tax imposed on sugary drinks raised USD 8 million in the first six months of implementation, which was channeled toward the purchase of cancer diagnostic and treatment equipment for public hospitals. Many other African countries have implemented taxes on sugar-sweetened beverages (SSBs). While these taxes can be a valuable tool for governments to bolster health financing, their effectiveness in promoting public health depends largely on how the revenue is allocated and used. A recent review found that only Uganda and South Africa designated some SSB tax revenue for health-related initiatives. Even in these cases, the allocations have been relatively modest, accounting for just 1–2% of the total collected [28]. To maximize the primary health benefits of SSB taxes, governments must establish clear frameworks for the utilization of these funds. This includes transparent allocation processes, targeted spending on evidence-based interventions, and regular monitoring and evaluation to ensure that the revenue is used effectively to improve public health.

#### 3.1.2. Donor Alignment

Development assistance is a major source of health funding in the region. Persistent aid fragmentation has hindered the ability of development assistance to effectively address health challenges in Africa, contributing to inefficiencies and health inequities [29]. To enhance the effectiveness of HIV service integration, donors should align their funding with local PHC priorities, ensuring that HIV-related funding reaches subnational health facilities. Subnational governments should lead in coordinating with donors to implement joint programming that ensures HIV funding is directed to local PHC structures. Additionally, pooled funding mechanisms, such as those implemented by Rwanda, can be effective tools to streamline resource allocation and enhance coordination. The country’s Health Sector Strategic Plan (HSSP) has played a crucial role in aligning donor priorities [30], ensuring funds are directed to the PHC level more cohesively and efficiently.

#### 3.1.3. Cost-Effectiveness Analysis

Conducting cost-effectiveness analyses can help identify the most efficient interventions for integrating HIV services into primary healthcare. Policymakers can make informed decisions about resource allocation by comparing the costs and benefits of different approaches. The hybrid approach can be better since it addresses the strengths and weaknesses of both sectoral and incremental analysis. Sectoral cost-effectiveness analysis has the potential to achieve significant efficiency benefits and is particularly well suited in situations where there are significant allocative inefficiencies in the current service provision. On the other hand, putting a thorough overhaul of the packages into reality can be difficult. When some new services have the potential to affect the long-term viability of the healthcare system, incremental cost-effectiveness analysis becomes more pertinent. It may support efficiency improvement, but its focus has typically been on new services while existing inefficiencies remain unchallenged. Investing in interventions with high cost-effectiveness ratios can maximize the impact of limited resources [31].

#### 3.1.4. Results-Based Financing

Results-based financing can incentivize the delivery of quality HIV services by linking funding to achieving specific performance targets. This approach can improve efficiency, accountability, and patient outcomes. However, it is essential to carefully design performance indicators and monitoring systems to achieve the desired results [32].

#### 3.1.5. Strengthening Health Financing Systems and Monitoring and Evaluation

Countries in SSA must invest in building robust health financing systems, including financial management, budgeting, and expenditure tracking. Financial planning and the management of health services should involve service delivery managers and finance managers [33]. They should also establish robust monitoring and evaluation systems (Mand E) to track the impact of investments and inform resource allocation decisions [34].

### 3.2. Strengthening the Health System for HIV Integration

#### 3.2.1. Policy Environment: Creating an Enabling Framework

A well-defined policy framework is essential for integrating HIV services into primary healthcare. Clear policies and regulations provide a roadmap for stakeholders, allocate resources, and establish accountability mechanisms. They also create a conducive environment for collaboration between different sectors and levels of government. Moreover, supportive policies can help to address stigma and discrimination, which are significant barriers to HIV prevention, testing, and treatment. The effective integration of HIV services into primary healthcare requires a supportive policy environment that facilitates implementation, promotes equity, and ensures sustainability. To create an enabling policy environment for HIV service integration, governments in SSA should conduct comprehensive policy assessments to assess existing policies and identify gaps and inconsistencies that hinder integration. They should create evidence-based policies that draw from thorough research and evidence to ensure effectiveness. All stakeholders, including government officials, healthcare providers, civil society organizations, and people living with HIV, should be engaged in policy development. There should be sufficient funding for policy development, implementation, and monitoring. Intersectoral collaboration should be strengthened to promote collaboration between health, education, social welfare, and other relevant sectors to address the social determinants of health. Governments in the region should invest in training and capacity building for policymakers, healthcare providers, and other stakeholders to effectively implement policies. These governments should establish clear accountability mechanisms and promote transparency in policy implementation. National-level policies should be accompanied by subnational-level implementation strategies that allow for the integration of HIV services into local PHC systems. Regularly monitoring and evaluating the implemented policies should be conducted to determine their impact and make necessary adjustments to improve outcomes.

South Africa’s National Health Insurance (NHI) policy has taken significant steps toward integrating HIV care into a universal health coverage framework. Early results from pilot districts indicate improved access to comprehensive primary healthcare services [35,36]. Rwanda’s inclusive policy environment, driven by its Health Sector Strategic Plan, has addressed the dual challenge of stigma and service integration by mandating HIV training across all primary care providers [37]. However, inconsistencies between policies and actual implementation persist in many other SSA countries, underscoring the need for robust accountability mechanisms and multi-sectoral collaboration.

#### 3.2.2. Human Resources for Health

A robust healthcare workforce is crucial for delivering quality HIV services. Subnational governments should prioritize increasing the number and distribution of healthcare workers trained in HIV care and prevention, particularly in rural and underserved areas. Policies should focus on increasing the number and distribution of healthcare providers, particularly in rural and underserved areas. Investing in training and capacity building is essential to equipping healthcare workers with the necessary skills to provide comprehensive HIV care. Furthermore, policies should address issues such as job satisfaction, retention, and workload management to improve the overall performance of the healthcare workforce [38]. Additionally, policies should promote task shifting and shared responsibilities among healthcare providers to optimize the use of human resources [39]. Malawi and Tanzania’s use of task shifting by training lower-cadre health workers to deliver antiretroviral therapy has significantly alleviated the burden on clinical staff [40]. This model and ongoing mentorship have expanded access to HIV services in rural areas. Similarly, Uganda’s Village Health Teams have been instrumental in bridging the gap between communities and formal healthcare systems. While these programs have shown promising outcomes, retaining trained personnel and addressing burnout remain critical challenges to long-term success.

#### 3.2.3. Service Delivery Models

Patient-centered care is positively associated with satisfaction with care and the physical and social well-being of patients with multiple morbidities in the primary care setting [41]. Flexible and patient-centered service delivery models are essential for effective HIV integration. Policies should support developing and implementing integrated service delivery points like community-based health centers and mobile clinics. A Community-Based HIV Care and Treatment (CB-HCT) program in a conflict-affected rural area of Yambio County, South Sudan, has effectively brought services closer to patients through decentralized care. This model combines peer-led support groups and mobile clinics to reach remote communities, achieving over 90% adherence rates among enrolled participants [42]. In Mozambique, the integration of HIV and maternal health services at antenatal clinics has resulted in improved HIV testing rates and better maternal health outcomes [43,44]. Expanding these models across diverse contexts requires adaptable strategies that account for local needs and resources.

#### 3.2.4. Health Information Systems

Strong health information systems are critical in the integration process. Technology tools, including electronic health records (EHRs), telehealth, clinical decision support (CDS), clinical registries, quality measure dashboards, and standards-based interoperability, can enable integration among various clinical disciplines and more generally improve the delivery of care so that it more readily addresses patient needs [45]. Nigeria’s adoption of the Lafiya Management Information System (LAMIS) has enhanced data collection and analysis, supporting more targeted interventions for HIV care [46]. Similarly, Kenya’s EMR system integration in rural health facilities has streamlined patient tracking and improved adherence to treatment [47]. Despite these advancements, gaps in interoperability and limited digital literacy among healthcare workers hinder the full potential of health information systems. Investing in scalable training programs and cross-platform compatibility can accelerate progress in this area.

#### 3.2.5. Monitoring and Evaluation

Robust Mand E systems are essential for tracking progress, identifying challenges, and measuring the impact of HIV service integration. Policies should establish clear indicators for monitoring and evaluation and mechanisms for data collection, analysis, and dissemination. The regular evaluation of program performance is crucial for identifying areas for improvement and ensuring that resources are used efficiently [48].

While significant strides have been made, several gaps remain in integrating HIV services into primary healthcare. Future efforts should prioritize scaling up proven interventions, such as community-based HIV care, across diverse settings while addressing structural challenges, including healthcare worker retention and infrastructure gaps in rural areas. Additionally, it is crucial to develop more robust frameworks for integrating HIV programs with other vertical health initiatives, such as tuberculosis and non-communicable diseases. Furthermore, evaluating the long-term sustainability of innovative financing mechanisms is essential to ensure continued progress in achieving comprehensive HIV service integration.

## 4. Implementation Strategies: Practical Steps for Integration

The successful integration of HIV services into primary healthcare requires a multi-faceted approach that addresses the needs of healthcare providers, patients, and communities. To successfully implement HIV service integration, the existing primary healthcare systems should be assessed to identify their strengths and weaknesses so that a comprehensive implementation plan that addresses the specific needs of the target population can be developed. However, the successful implementation of these steps requires the development of sustainable financing mechanisms to support the long-term implementation of HIV service integration. In this section, we draw on existing research, reviews, and expert opinions to outline practical strategies for strengthening the primary healthcare workforce, optimizing service delivery, and leveraging technology to enhance HIV care. Table 2 presents specific implementation strategies. These strategies focus on practical steps for integrating HIV services, involving workforce strengthening, service delivery optimization, and technology use.

### 4.1. Strengthening the Primary Healthcare Workforce

A well-trained and motivated healthcare workforce is essential for delivering quality HIV services. Investing in the capacity building of primary healthcare providers is crucial for ensuring effective integration.

#### 4.1.1. Training and Capacity Building

Comprehensive training programs should be developed to equip healthcare providers with the knowledge and skills necessary to provide HIV prevention, testing, treatment, care, and support services. This includes training in clinical management, counseling, adherence support, and patient education. Regular refresher training and updates on new guidelines are essential to maintaining competency. Additionally, mentorship and coaching programs can support healthcare providers in developing their skills and confidence [49]. 

Programs like the Integrated Management of Childhood Illness (IMCI) and Essential Care for Every Infant, Child, and Adolescent (ECECIA), which incorporate HIV components, have demonstrated success in improving care quality for pediatric and adolescent populations [50]. For example, in Mozambique, these programs enhanced providers’ skills in HIV counseling and diagnosis, leading to improved pediatric ART initiation [51]. Regular refresher courses and updates aligned with evolving guidelines, alongside mentorship initiatives such as South Africa’s “on-the-job coaching” model, can build providers’ confidence and competency [52].

#### 4.1.2. Job Satisfaction and Retention

Creating a supportive work environment is crucial for retaining healthcare providers in primary care settings. This includes offering competitive salaries, opportunities for professional development, and adequate workload management. Implementing effective supervision and mentorship programs can also contribute to job satisfaction [53]. Innovative strategies such as task shifting and job redesign can be explored to address the challenge of healthcare worker shortages, particularly in rural areas. Community health workers can be trained to deliver basic HIV services, thereby relieving the workload of healthcare providers and increasing access to care [54].

### 4.2. Optimizing Service Delivery

Patient-centered care, integrated service delivery models, and community engagement are key to successful HIV service integration.

#### 4.2.1. Patient-Centered Care

Adopting a patient-centered approach is essential for improving care quality and patient satisfaction. This involves empowering patients to make informed decisions about their health, building strong patient–provider relationships, and addressing patients’ needs and preferences. Implementing patient-reported outcome measures can help to monitor patient experiences and identify areas for improvement [41]. Peer support programs can be effective in providing emotional support and practical assistance to people living with HIV. Peer educators can share their experiences, reduce stigma, and treatment adherence [55]. Implementing patient-reported outcome measures in Malawi improved adherence rates and revealed areas requiring enhanced support. Peer-led interventions, such as Zambia’s adherence clubs, reduced stigma and improved treatment adherence by creating safe spaces for sharing experiences [56].

#### 4.2.2. Integrated Service Delivery Models

Integrating HIV services with other primary healthcare services can improve efficiency, reduce costs, and enhance patient convenience. Offering a comprehensive package of services, including maternal and child health, family planning, and chronic disease management, can increase the utilization of HIV services [7]. 

Community-based HIV testing and counseling services can increase access to care for people who may be reluctant to visit a health facility. Mobile clinics can also be used to reach underserved populations [57]. For instance, Ethiopia’s Health Extension Program incorporates maternal and child health with HIV care, providing a one-stop model that enhances service utilization [58]. Similarly, mobile clinics in Tanzania brought testing and counseling services to underserved rural populations, significantly increasing HIV diagnosis and linkage to care rates.

#### 4.2.3. Community Engagement

Strong community engagement is essential for successful HIV service integration. Community-based organizations can play a vital role in raising awareness about HIV, reducing stigma, and mobilizing community support. Community health workers can serve as a bridge between the health system and the community, facilitating access to care and promoting treatment adherence [7]. 

Involving people living with HIV in program planning and implementation can help to ensure that services are responsive to their needs. Community advisory boards can provide valuable input on program design and implementation [7]. Governments in SSA should, therefore, collaborate with community-based organizations, civil society, and other stakeholders to build a strong support network. In Zimbabwe, community health workers facilitated treatment adherence by conducting home visits and linking patients to support groups. Additionally, community advisory boards in Eswatini effectively influenced program designs, ensuring they were culturally sensitive and responsive to local needs [59].

### 4.3. Leveraging Technology

Digital health technologies hold immense potential for enhancing the efficiency and effectiveness of HIV service delivery in SSA. EHRs, for instance, can streamline patient registration, track treatment adherence, and generate data for program monitoring [60]. Mobile health applications can provide patients with reminders for medication, appointment scheduling, and access to educational materials [61]. Telemedicine can expand access to specialized care in remote areas [62]. Several studies have demonstrated the positive impact of these technologies on HIV service delivery. For example, EHRs reduced patient wait times in Kenya and improved HIV treatment adherence [63]. This enhances patient care and frees healthcare provider time for more complex tasks, thereby improving overall healthcare system efficiency. Lessons learned have been applicable beyond HIV/AIDS to include primary care, chronic disease management, and community-based health screening and disease prevention programs.

#### 4.3.1. Digital Health Tools

Mobile health (mHealth) applications can be used to support patient self-management, medication adherence, and appointment reminders. Electronic health records can facilitate the exchange of patient information between healthcare providers and improve the quality of care. Telemedicine can be used to provide remote consultations and support, especially in areas with limited healthcare resources [64]. Digital tools can also be used to collect data on HIV service utilization, patient outcomes, and program performance. These data can be used to inform decision-making and improve program effectiveness [65].

#### 4.3.2. Data Management and Utilization

Effective data management is essential for using digital health tools to their full potential. Robust data systems are needed to collect, store, analyze, and share health information. Data quality and privacy must be ensured to protect patient confidentiality. Data analysis can be used to identify trends, monitor program performance, and inform evidence-based decision-making. Data visualization tools can be used to communicate findings effectively to policymakers, program managers, and healthcare providers [66]. Therefore, governments in SSA should invest in data management and analysis to inform program planning, implementation, and evaluation.

### 4.4. Monitoring and Evaluation: Measuring Progress and Impact

Effective Mand E systems are crucial for tracking the progress of HIV service integration, identifying challenges, and informing decision-making. A robust M and E system provides valuable insights into the performance of HIV service integration programs. It helps to assess the reach and coverage of HIV services, measure the quality of care provided, evaluate the impact of interventions on patient outcomes, identify cost-effective approaches, and inform resource allocation and policy decisions.

To effectively monitor and evaluate HIV service integration, a comprehensive set of indicators should be used. These indicators can be grouped into several categories: service coverage, quality of care, patient outcomes, and cost-effectiveness. Service coverage indicators include the proportion of people living with HIV who are aware of their status, on ART, and virally suppressed. In contrast, quality of care indicators include adherence rates and retention in care rates. Indicators related to patients’ outcomes include morbidity rates and mortality rates. Cost-effectiveness analysis can be used to assess the value for money of different HIV service integration strategies.

## 5. Conclusions

In this perspective, we provide actionable recommendations for advancing the integration of HIV services into primary healthcare in sub-Saharan Africa. Drawing on both empirical data and real-world experiences, we emphasize the need for systemic transformations to sustain HIV gains and ensure long-term progress beyond 2030. Strengthening the primary healthcare workforce through comprehensive training, mentorship, and enhancing job satisfaction is critical. This will help ensure the provision of high-quality, accessible HIV services, addressing key challenges such as healthcare worker shortages and the uneven distribution of skilled personnel. Additionally, the integration of HIV services with maternal and child health, family planning, and chronic disease management is essential for creating comprehensive service delivery frameworks that ensure equitable and continuous access to care. This patient-centered approach not only improves outcomes for people living with HIV but also enhances service utilization across populations, particularly in marginalized communities. Efforts to leverage digital health technologies, including mobile health applications, electronic health records, and telemedicine, should be ramped up. These tools have demonstrated significant potential in improving efficiency, enhancing patient adherence to treatment, and optimizing resource use, particularly in resource-constrained settings. They also provide valuable data for program monitoring and evidence-based decision-making, which are vital for adapting to the evolving challenges of the epidemic. Furthermore, the role of community engagement cannot be overstated. Ensuring the active participation of people living with HIV and other key populations in the planning, implementation, and evaluation of HIV services strengthens the responsiveness of programs and fosters local ownership. Building strong community-based support networks and empowering local leadership are essential for sustainable HIV responses. Finally, integrating HIV services into primary healthcare requires a multi-faceted approach that addresses not only the structural and financial challenges but also the human and community factors that contribute to the success of HIV programs.

## Figures and Tables

**Table 1:** Recommendations for integrating HIV services into PHC.

Recommendation Area	Specific Actions
Policy and Governance	- Align national strategies to include HIV services within PHC frameworks.
	- Strengthen leadership and establish coordination mechanisms for integration.
	- Ensure alignment with global health goals (e.g., UHC, SDGs).
Workforce Capacity Building	- Train PHC providers on HIV testing, counseling, and ART protocols.
	- Incorporate HIV modules into healthcare worker training curricula.
	- Implement task shifting to delegate routine HIV services to CHWs.
Service Delivery	- Develop one-stop shops co-locating HIV and other PHC services.
	- Include HIV services in ANC, family planning, and chronic disease management.
	- Strengthen proactive community outreach for HIV education and testing.
Health Information Systems	- Integrate HIV data into PHC health management information systems.
	- Ensure interoperability between HIV-specific and broader PHC data systems.
	- Use digital tools for patient tracking and adherence monitoring.
Resource Allocation	- Ensure consistent supply of HIV diagnostics, ART, and prevention tools.
	- Integrate HIV and PHC supply chains to improve efficiency and reduce costs.
Financial Sustainability	- Allocate specific HIV funding within PHC budgets.
	- Leverage public–private partnerships and donor support.
Community Engagement	- Address stigma and discrimination through community campaigns.
	- Partner with local leaders and groups to build trust and advocate for integration.
	- Support PLHIV as peer educators for counseling and awareness.
Monitoring and Quality	- Develop indicators to measure integrated HIV-PHC services (e.g., ART adherence, testing rates).
	- Conduct regular facility audits and patient feedback sessions.
	- Use operational research to identify barriers and scalable solutions.
Tailored Services	- Design targeted interventions for key populations (e.g., adolescents, pregnant women).
	- Strengthen integration with PMTCT programs within PHC.
Advocacy and Political Will	- Engage policymakers to prioritize and fund HIV-PHC integration efforts.
	- Build public awareness of the benefits of integrated services.

**Table 2:** Practical implementation strategies for integrating HIV services into primary healthcare.

Implementation Strategy	Specific Actions
Workforce Strengthening	- Develop and implement comprehensive HIV training programs for primary healthcare providers.
	- Establish mentorship and coaching programs to support skill development.
	- Introduce job redesign and task shifting to delegate basic HIV care tasks to community health workers (CHWs).
	- Create incentives for healthcare workers in underserved areas, including salary supplements and professional development opportunities.
Service Delivery Models	- Implement integrated service delivery models, combining HIV services with maternal and child health, family planning, and chronic disease management.
	- Promote community-based HIV testing and counseling through outreach and mobile clinics.
	- Develop “one-stop” HIV services co-located with other primary care services for ease of access.
Patient-Centered Care	- Adopt patient-reported outcome measures (PROMs) to monitor and enhance patient satisfaction and adherence.
	- Establish peer support programs to reduce stigma and provide emotional support.
	- Empower patients with information to make informed decisions about their care, integrating counseling and educational tools.
Technology Utilization	- Integrate electronic health records (EHRs) for streamlined patient tracking and adherence monitoring.
	- Implement mobile health (mHealth) applications to remind patients of appointments and medications and provide educational content.
	- Expand telemedicine services to provide remote consultations and care in underserved areas.
	- Utilize digital platforms for real-time data collection, analysis, and decision-making to optimize service delivery.
Community Engagement	- Engage community health workers to support HIV care access and adherence through home visits and community mobilization.
	- Foster partnerships with community-based organizations to reduce stigma and increase awareness.
	- Involve people living with HIV in the design and implementation of services, ensuring they are culturally sensitive and meet community needs.
Monitoring and Evaluation	- Develop and track key indicators such as ART adherence, treatment retention, and community engagement.
	- Use data visualization tools to communicate performance data to stakeholders.
	- Conduct regular audits of service delivery models to identify barriers and areas for improvement.
Sustainable Financing	- Identify long-term funding mechanisms for integrating HIV services, including government funding, public–private partnerships, and donor contributions.
	- Implement cost-effective strategies for HIV service delivery and resource allocation to ensure sustainability.
Data Management and Utilization	- Invest in robust data systems to capture, store, and analyze HIV service delivery and outcomes.
	- Ensure data privacy and security while improving interoperability between HIV and broader PHC data systems.
	- Leverage data analytics to track trends and inform future service delivery strategies.

## Data Availability

Not applicable.
